# Quercetin regulates the sestrin 2-AMPK-p38 MAPK signaling pathway and induces apoptosis by increasing the generation of intracellular ROS in a p53-independent manner

**DOI:** 10.3892/ijmm.2014.1658

**Published:** 2014-02-13

**Authors:** GUEN TAE KIM, SE HEE LEE, JONG IL KIM, YOUNG MIN KIM

**Affiliations:** Department of Biological Sciences, College of Life Science and Nanotechnology, Hannam University, Yuseong-gu, Daejeon 305-811, Republic of Korea

**Keywords:** quercetin, reactive oxygen species, sestrin 2, mitochondria, apoptosis

## Abstract

The induction of apoptosis in cancer cells is a therapeutic strategy for the treatment of cancer. In the present study, we investigated the regulatory mechanisms responsible for quercetin-induced apoptosis, mamely the increased expression of sestrin 2 and the activation of the 5′ AMP-activated protein kinase (AMPK)/p38 MAPK signaling pathway. Our results revealed that quercetin induced apoptosis by generating the production of intracellular reactive oxygen species (ROS) and increasing the expression of sestrin 2. The induction of apoptosis by quercetin occurred through the activation of the AMPK/p38 signaling pathway and was dependent on sestrin 2. However, the silencing of sestrin 2 using small interfering RNA (siRNA) targeting sestrin 2 revealed that quercetin did not regulate AMPK or p38 phosphorylation in the cells in which sestrin 2 was silenced. On the other hand, it has been previously reported that sestrin 2 expression is not dependent on p53 expression under hypoxic conditions, whereas DNA damage is dependent on p53. We demonstrate that the increase in the expression of sestrin 2 by quercetin-generated intracellular ROS is p53-independent. The increased expression of sestrin 2 induced apoptosis through the AMPK/p38 signaling pathway in the HT-29 colon cancer cells, which are p53 mutant, treated with quercetin. Thus, our data suggest that quercetin induces apoptosis by reducing mitochondrial membrane potential, generating intracellular ROS production and increasing sestrin 2 expression through the AMPK/p38 pathway. In addition, p53 is not a necessary element for an apoptotic event induced by sestrin 2.

## Introduction

The incidence of cancer continues to increase largely due to aging and the increased adoption of cancer-causing behavior, particularly smoking and obesity. Specifically, colorectal cancer is the third most common cancer in males and the second in females, with over 1.2 million new cases and 608,700 deaths in 2008 (1). Histologically, colorectal cancer incidence rates are higher in western countries, such as New Zealand, Europe and North America than in Asian countries. However, the incidence rates are rapidly increasing in several countries within Eastern Asia, such as Japan, China and Singapore (2). Such trends are thought to reflect a change in dietary patterns (3). For this reason, the interest in alternative medicine for the prevention and treatment of colorectal cancer has increased and research of the effects of various food extracts on colorectal cancer is in progress. Among food extracts, quercetin (3,3′,4′,5,7-pentahydroxyflavone), which is an extracted compound from green tea and red onion, has been reported as being able to prevent a number of types of cancer, including colorectal cancer (4). In tumor cells, quercetin exerts a direct pro-apoptotic effect by regulating caspase-3, -6 and -8, and the 5′ AMP-activated protein kinase (AMPK)/cyclooxygenase-2 (COX-2) signaling pathway. In addition, it can block the growth of cancer cells at different phases of the cell cycle by controlling transcription factors, such as p53 (5–7).

Sestrin 2 is a downstream effector of p53 and is involved in the regulation of cell viability in a variety of cellular stresses. Sestrin 2 expression is induced upon DNA damage, such as UV-irradiation, dependent on p53, and oxidative stress, such as hypoxia, independent of p53. Previous studies have indicated that sestrin 2 expression in cancer cells suppresses cell growth and proliferation by leading to the negative control of mTOR through AMPKα1 phosphorylation (8,9). In addition, sestrin 2 can function as an antioxidant agent under low concentrations of hydrogen peroxide. This leads to the regeneration of peroxiredoxin for the reduction of hydrogen peroxide levels and the maintenance of cell viability through the nuclear factor erythroid-derived 2-related factor 2 (Nrf-2)/ARE signaling pathway; however, under high concentrations of hydrogen peroxide, sestrin 2 induces apoptosis through the p53 signaling pathway (10–13).

AMPK and serine/threonine protein kinase, participate in an energy-sensing cascade that responds to the deletion of adenosine triphosphate (ATP). AMPK is activated by various upstream factors, such as liver kinase B1 (LKB1) or Ca(2+)/calmodulin-dependent protein kinase kinase (CaMKK), inhibited by mTOR, and leads to apoptosis by the activation of Tuberous sclerosis 2 (TSC2) (14–16). Previous studies have indicated that the activation of AMPK by quercetin-generated reactive oxygen species (ROS) induces apoptosis through the apoptosis signal-regulating kinase 1 (ASK1)/p38 MAPK pathway in MCF-7 breast cancer cells (17). In addition, activated AMPK by UV radiation or hydrogen peroxide has been shown to lead to cell death through p38 MAPK activation (18). A number of studies have shown the involvement of AMPK upstream pathways in a variety of conditions, such as high glucose concentrations, ischemia and calcium ion-dependent signals, or extracellular stress; however, the activation of AMPK by any upstream signaling pathway under stress-induced conditions, such as hydrogen peroxide or flavonoid-generated ROS is not yet fully understood (19–21).

In the present study, we demonstrate that quercetin promotes the generation of intracellular ROS and induces apoptosis by decreasing mitochondrial membrane potential through the AMPK/p38 MAPK pathway and that these effects are dependent on sestrin 2 expression. Moreover, the activation of the sestrin 2/AMPK/p38 pathway induced by the quercetin generation of ROS occurred independently of p53.

## Materials and methods

### Reagent

Quercetin, N-acetylcysteine (NAC), 3-(4,5-dimethylthiazol-2-yl)-2,5-diphenyltetrazolium bromide (MTT), dichloro-dihydro-fluorescein diacetate (DCFH-DA) and 3,3-dihexyloxacarbocyanine iodide (DiOC_6_) were all purchased from Sigma-Aldrich (St. Louis, MO, USA). SB203580 (p38 MAPK inhibitor) and pifithrin-α were purchased from Calbiochem (San Diego, CA, USA). The FITC-Annexin V apoptosis detection kit was obtained from BD Pharmingen (San Diego, CA, USA). Specific antibodies that recognized phosphorylated (p-)AMPKα1, AMPKα1, Bax, caspase-3, cytochrome *c* and β-actin are obtained from Cell Signaling Technology (Beverly, MA, USA) and sestrin 2 was purchased from Proteintech (Chicago, IL, USA). p-p38 MAPK was purchased from Signalway Antibody LLC (College Park, MD, USA).

### Cell culture

HCT116 and HT-29 colon cancer cells were obtained from the American Type Culture Collection (ATCC; Rockville, MD, USA). The cells were grown in RPMI-1640 medium (HyClone, Waltham, MA, USA) containing 10% fetal bovine serum (HyClone) and 1% antibiotics (100 mg/l streptomycin and 100 U/ml penicillin) at 37°C in a 5% CO_2_ atmosphere. The cells were suspended by Trypsin-EDTA (HyClone) and separated at 1.5×10^5^/ml per plate, every 48 h.

### Detection of intracellular ROS by fluorescence microscopy

The cells were seeded 1×10^5^/ml in a 12-well plate with coverglasses. Following treatment for the indicated periods of time and doses at 37°C in a 5% CO_2_ atmosphere, the cells were incubated with 10 μM of DCFH-DA for 30 min and fixed with 3.7% formaldehyde for 20 min. The cells were washed with PBS twice and fluorescence was detected under a fluorescence microscope (Carl Zeiss, Thornwood, NY, USA).

### Measurement of intracellular ROS levels

The cells were seeded 1×10^6^/ml in 100-mm plate and incubated for 24 h. Following incubation, the cells were treated with the test compound for 6 h at 37°C in a 5% CO_2_ atmosphere. The cells were then incubated with 40 μM of DCFH-DA for 30 min and harvested by trypsinization, collected by centrifugation, washed with PBS twice, and resuspended in PBS. The fluorescence intensity was analyzed using a flow cytometer (BD Biosciences, Frankline Lakes, NJ, USA).

### Cell proliferation assay (MTT assay)

The cells were seeded at 4,000/ml each well in a 96-well plate, and incubated for 24 h. Following incubation, the cells were treated with the test compound and then incubated at 37°C in a 5% CO_2_ atmosphere. After 24 h, the cells were incubated with 20 μl MTT (5 mg/ml with PBS) solution for 1 h. The optical densities of the solution in each well were determined using a microplate reader (Bio-Rad Laboratories, Inc., Tokyo, Japan) at 595 nm.

### Determination of apoptosis by Annexin V/PI staining

The cells were seeded at 1×10^6^/ml in 100-mm plate and incubated for 24 h. Following incubation, the cells were treated with the test compound for 24 h at 37°C in a 5% CO_2_ atmosphere. Total cells were harvested by trypsinization, collected by centrifugation, washed with PBS, and resuspended in binding buffer. Cells were stained with Annexin V and PI for 15 min. Fluorescence intensity were analyzed using a flow cytometer (BD Biosciences).

### Determination of apoptosis by Hoechst 33342 staining

The cells were seeded at 1×10^4^/ml in a 12-well plate with coverglasses. Following treatment at the indicated doses, the cells were incubated with 10 μM Hoechst 33342 for 30 min and fixed with 3.5% formaldehyde for 20 min. The cells were then washed twice with PBS, and fluorescence was measured using a fluorescence microscope (Carl Zeiss).

### Measurement of mitochondrial membrane potential

The cells were seeded at 1×10^6^/ml in a 100-mm plate and incubated for 24 h. Following incubation, the cells treated with the test compound for 24 h at 37°C in a 5% CO_2_ atmosphere. Total cells were harvested by trypsinization, collected by centrifugation, washed with PBS, and fixed with 70% ethanol. Cell were incubated with 100 ng/ml of DiOC_6_ for 15 min at room temperature before being analyzed under a flow cytometer (BD Biosciences).

### Mitochondrial and cytosolic fractions

We used the Mitochondria/Cytosol Fractionation kit (Abcam plc, Cambridge, UK). The cells were seeded at 1×10^6^/ml in 100-mm plate and incubated for 24 h. Following incubation, the cells were treated with the test compound for 24 h at 37°C in a 5% CO_2_ atmosphere. Total cells were harvested by trypsinization, collected by centrifugation, washed with PBS, and homogenized in ice-cold cytosol extraction buffer mix containing DTT and protease inhibitor using a homogenizer. The homogenates were centrifuged at 3,000 rpm for 10 min at 4°C and supernatants were collected. The supernatants were centrifuged at 13,000 rpm for 30 min at 4°C and the collected supernatants for cytosolic proteins and pellets were resuspended with ice-cold mitochondrial extraction buffer containing DTT and protease inhibitor for mitochondrial proteins.

### Transient transfection with small interfering RNA (siRNA)

siRNA was purchased from Dharmacon (Chicago, IL, USA). For transient transfection, the cells were seeded 5×10^3^/ml on a 6-well plate with antibiotics-free medium. Following incubation overnight, targeting siRNA was transfected using DharmaFECT1 transfection reagent (Dharmacon) according to the manufacturer’s instructions. Following incubation for 72 h, the cells were treated with quercetin for the indicated periods of time.

### Western blot analysis

The cells were seeded at 1×10^5^/ml in a 6-well plate and incubated for 24 h. Following incubation, the cells were treated with the test compound for 6 h at 37°C in a 5% CO_2_ atmosphere. The cells were then rinsed twice with ice-cold PBS and scraped with lysis buffer (50 mM Tris-HCl pH 8.0, 150 mM NaCl, 1% NP-40, 0.5% sodium deoxycholate, 1 mM PMSF) and subjected to western blot analysis. The primary antibody was then added following by overnight incubation at 4°C; following the addition of the secondary antibody, the cells were reacted for 75 min at room temperature with gentle agitation.

### Statistical analysis

Cell viability was statistically analyzed using an unpaired t-test (SPSS Inc.; Chicago, IL, USA). A value of P<0.05 was considered to indicate a statistically significant difference.

## Results

### Quercetin generates intracellular ROS production in HCT116 colon cancer cells

**Figure 1:**
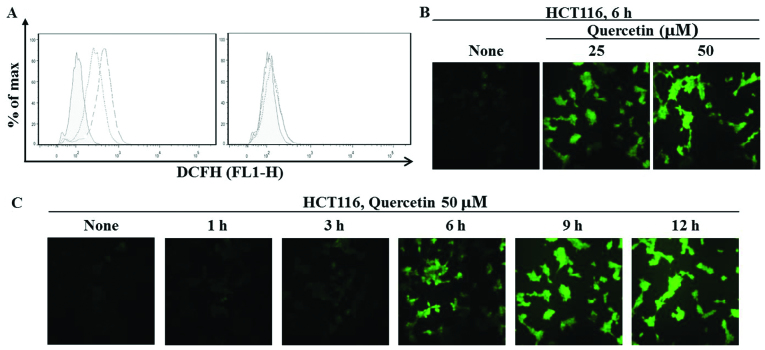
(A) Quercetin increased intracellular reactive oxygen species (ROS) production. Cells were treated with the indicated concentrations of quercetin for 6 h (left panel), pre-treated with 5 mM N-acetylcysteine (NAC) for 30 min, and then exposed to quercetin (right panel). After 6 h, teh cells were treated with 40 μM dichloro-dihydro-fluorescein diacetate (DCFH-DA) for 30 min, and fluorescence intensity was measured using a flow cytometer. Dashed line, quercetin 25 μM; dotted line, quercetin 50 μM. (B and C) In addition, cells were treated with the indicated concentrations quercetin for the indicated periods of time and fluorescence was detected using a fluorescence microscope.

To examine whether quercetin promotes the generation of ROS in HCT116 colon cancer cells, we measured the intracellular ROS levels following treatment of the cells with quercetin (25 μM or 50 μM) for 6 h. As shown in Fig. 1A, quercetin increased ROS levels at the indicate concentrations (left panel). These effects were completely blocked by combined treatment with NAC, a ROS scavenger (right panel). We also observed intracellular ROS levels under a fluorescence microscope following staining with DCFH-DA. The quercetin-induced the generation of ROS continuously in a dose and time-dependent manner (Fig. 1B and C).

### Quercetin suppresses cell proliferation and induces apoptosis

**Figure 2:**
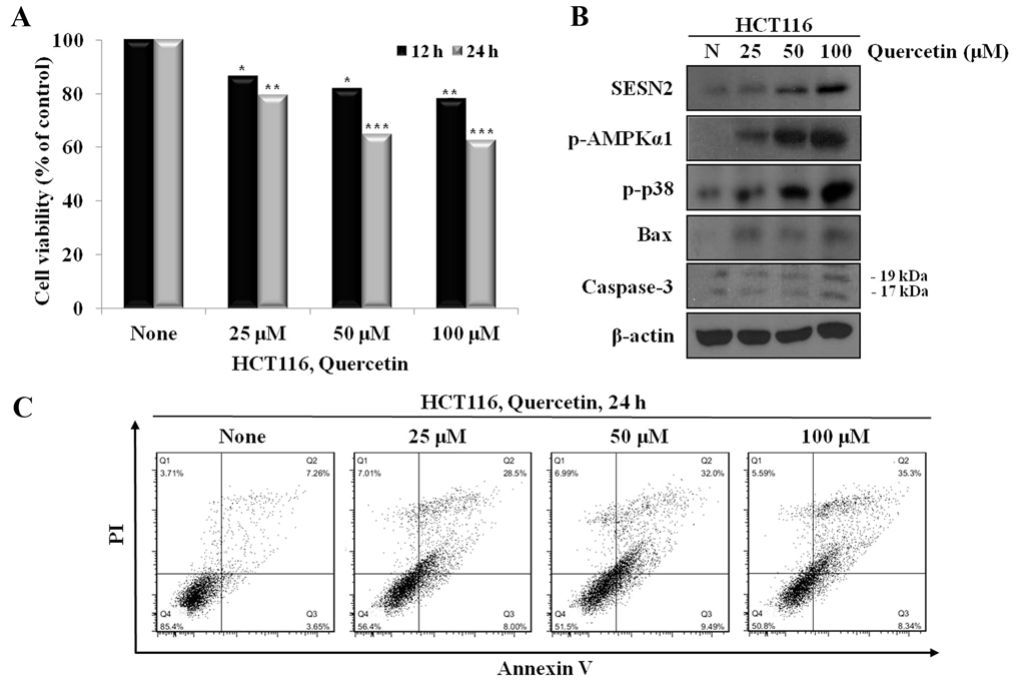
(A) Cell viability was measured by MTT assay. ^*^P<0.05, ^**^P<0.01 and ^***^P<0.001 (each experiment, n=3). (B) Cells were treated with the indicated concentrations of quercetin for 6 h. The expression of sestrin 2 and the activation of AMPKα1 and p38 were analyzed by western blot analysis. (C) Cells were treated with the indicated concentrations of quercetin for 24 h. Cells were stained with Annexin V/PI and fluorescence intensity was measured using a flow cytometer. SESN2, sestrin 2.

We investigated the anti-proliferative and apoptotic effects of quercetin through the increase in intracellular ROS. For this reason, we treated the cells with quercetin (25, 50 and 100 μM) for 24 h, and the viability and apoptosis of the cells were then examined. The cells treated with quercetin showed a decrease in viability and an increase in the number of Annexin V-positive cells in a dose-dependent manner (Fig. 2A and C).

### Quercetin regulates the expression of sestrin 2 and AMPK, and p38 activation

To examine the mechanisms through which quercetin induces apoptosis, we first examined the mechanisms through which quercetin increases sestrin 2 expression and activates AMPKα1 and p38. We analyzed the changes in the levels of sestrin 2, p-AMPKα1 and p-p38, as well as apoptosis-related proteins, such as Bax and caspase-3 following treatment with quercetin at different concentrations by western blot analysis. Our results revealed that quercetin markedly increased the expression of sestrin 2 and activated AMPK and p38 in a dose-dependent manner. In addition, we also observed the increased expression of Bax and the cleavage of caspase-3 (Fig. 2B).

### Quercetin modulates the expression of sestrin 2 and AMPK, and the activation of p38 through the generation of intracellular ROS

**Figure 3:**
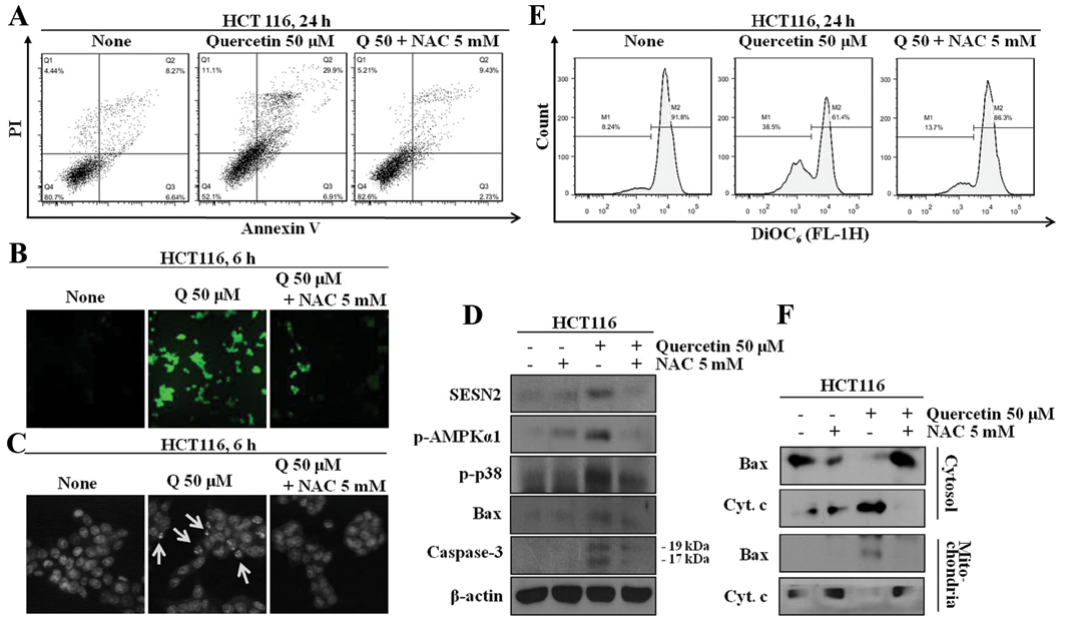
Cells were pretreated with 5 mM N-acetylcysteine (NAC) for 30 min and then exposed to quercetin for the indicated periods of time. (A) Cells were stained with Annexin V/PI and fluorescence intensity was measured using a flow cytometer. (B) cells were treated with 40 μM dichloro-dihydro-fluorescein diacetate (DCFH-DA) for 30 min, and fluorescence was detected using a fluorescence microscope. (C) Cells were treated with 10 μM Hoechst 33342 for 30 min, and fluorescence was detected using a fluorescence microscope. Arrows indicate apoptotic bodies, which were DNA fragments produced when apoptosis occurred. (D) The expression of sestrin 2 and the activation of AMPKα1, p38 were analyzed by western blot analysis. (E) Cells were stained with 3,3-dihexyloxacarbocyanine iodide (DiOC_6_) and fluorescence intensity was measured using a flow cytometer. (F) Cytochrome *c* in the mitochondrial/cytosolic fraction and Bax protein levels were analyzed by western blot analysis. Q, quercetin; SESN2, sestrin 2; Cyt. c, cytochrome *c*.

To determine whether the quercetin-induced increase in the expression of sestrin-2 and AMPK and the induction of apoptosis are involved in the increase in intracellular ROS levels, the cells wer co-treated with NAC and an quercetin; the proteins levels and the number of and Annexin V-positive cells were then determined. The cells co-treated with quercetin and NAC displayed decreased expression levels of sestrin 2, AMPK and p38 phosphorylation. The quercetin-treated group displayed increased apoptotic cell death through the regulation of mitochondrial membrane potential, leading to the secretion of cytochrome *c*, which is a marker protein for apoptosis, from the mitochondria to the cytosol (Fig. 3).

### Sestrin 2 is an important element for the induction of apoptosis and the activation of the AMPK/p38/BAX signaling pathway

**Figure 4:**
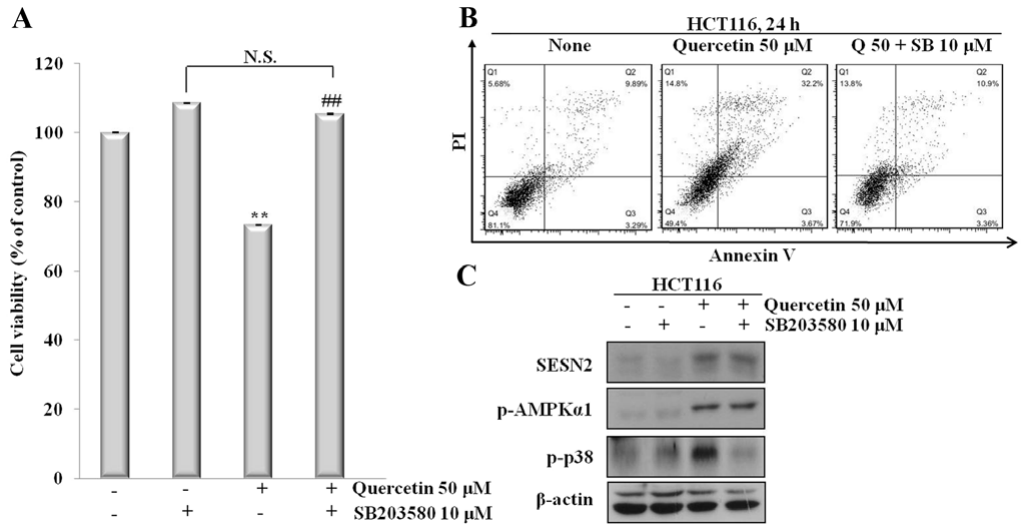
Cells were pre-treated with 10 μM SB203580 for 30 min and then exposed to quercetin for the indicated periods of time. (A) Cell viability was measured by MTT assay. ^**^P<0.01 compared to control; ^##^P<0.01 compared to the 50 μM quercetin-treated group. N.S., not significant (each experiment, n=3). (B) Cells were stained with Annexin V/PI and fluorescence intensity was measured using a flow cytometer. (C) The expression of sestrin and the activation of AMPKα1 and p38 were analyzed by western blot analysis. Q, quercetin; SESN2, sestrin 2.

**Figure 5:**
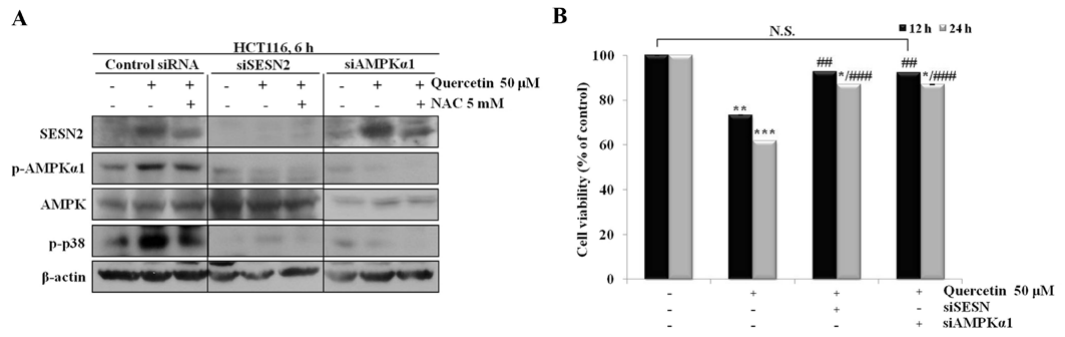
(A) Cells were transfected with sestrin 2 or AMPKα1 small interfering RNA (siRNA) using DharmaFECT and treated with 50 μM quercetin for 6 h after being pre-treated with N-acetylcysteine (NAC) 5 mM for 30 min. The protein levels of sestrin 2, p-AMPKα1, AMPKα1 and p-p38 were then examined by western blot analysis. (B) Cell viability was measured by MTT assay. ^*^P<0.05, ^**^P<0.01 and ^***^P<0.001 compared to control, ^##^P<0.01, and ^###^P<0.001 compared to the 50 μM quercetin-treated group. N.S., not significant (each experiment, n=3). SESN2, sestrin 2.

To determine whether the quercetin-induced apoptosis was dependent on sestrin 2 expression, and whether the activation of the AMPK/p38 signaling pathway was involved, we co-treated the cells with quercetin and SB203580, a p38 inhibitor. The cells were then analyzed for apoptotic cell death by Annexin V/PI staining and the proteins levels were determined by western blot analysis. Co-treatment with the inhibitor led to reduced cell death; however, the cells treated with quercetin displayed increased apoptotic cell death. In addition, co-treatment with quercetin and SB203580 induced an increase in the expression of sestrin 2 and AMPK phosphorylation (Fig. 4). Of note, quercetin did not regulate the AMPK/p38 signaling pathway and did not reduce cell viability when sestrin 2 was silenced using siRNA (Fig. 5).

### Sestrin 2 is expressed and induces apoptosis in p53-negative cells

**Figure 6:**
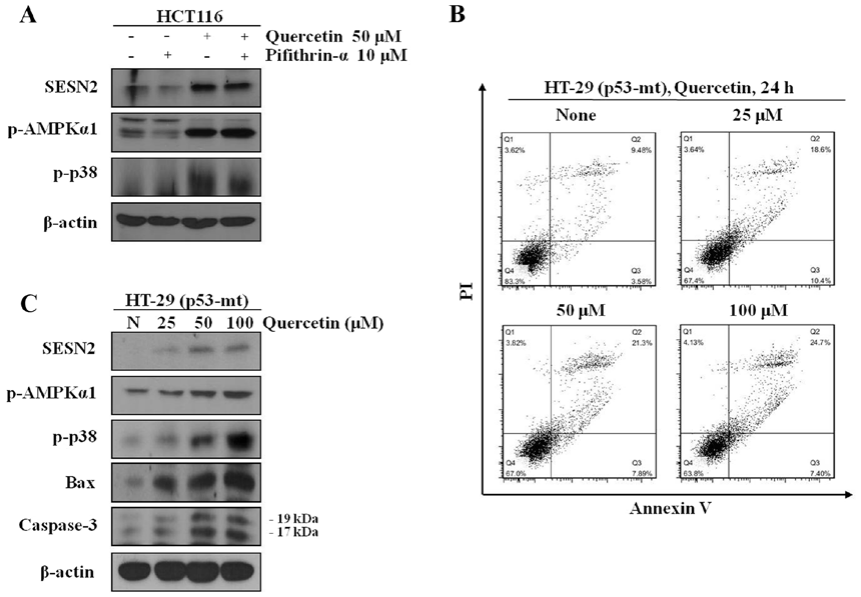
(A) HCT116 colon cancer cells were treated with the indicated concentrations of quercetin after being pre-treated with 10 μM pifithrin-α for 30 min. (B) HT-29 cells [p53-mutant (mt)] were treated with the indicated concentrations of quercetin for 24 h. Finally, the cells were stained with Annexin V/PI and their fluorescence intensity was measured using a flow cytometer. (C) HT-29 colon cancer cells were treated with the indicated concentrations of quercetin in for 6 h. The expression of sestrin 2 and the activation of AMPKα1 and p38 were analyzed by western blot analysis. SESN2, sestrin 2.

To examine the expression of sestrin 2 independent of p53, we co-treated the cells with quercetin and pifithrin-α, a p53-dependent transactivity inhibitor. Co-treatment with quercetin and pifithrin-α led to an increase in the expression of sestrin 2, AMPK and p38 phosphorylation. In addition, our results revealed that sestrin 2 expression levels, as well as those of AMPK and p38 phosphorylation were increased in the HT-29, which are p53 mutant cells. Moreover, quercetin induced apoptotic cell death in the HT-29 cells (Fig. 6).

## Discussion

The incidence of colorectal cancer has increased due to changes in dietary patterns. For this reason, there has been an increased interest in the effects of food extracts on the prevention and treatment of colorectal cancer. These foods extracts induce apoptosis and cell cycle arrest through the regulatkion of intracellular protein signals in cancer cells, leading to abnormal cell proliferation. Various foods extracts have been used in chemotheraphy experiments. This is particularly the case with quercetin, which is a polyphenolic compound extracted from red onion and green tea, and is known to have diverse pharmacological activities, including anticancer, anti-inflammatory and anti-proliferative activities (4,22). Recently, a study found that quercetin generates intracellular ROS and induces apoptosis through controlling the AMPK/ASK1/p38 pathway in MCF-7 breast cancer cells (17). Moreover, in a previous study, when HT-29 colon cancer cells were treated with quercetin, apoptosis was induced through the regulation of the AMPK/COX-2 pathway (6). It has been suggested that quercetin induces apoptosis through the activation of AMPK. However, the activation of AMPK by any upstream factors following treatment with quercetin is not clear. According to certain studies, the transcription of p53 induces AMPK phosphorylation; however, these results cannot explain the phosphorylation of AMPK in p53 mutant cells (23,24). However, as previously demonstrated, a specific type of stress, such as oxidative stress or DNA damage stimulates sestrin 2 transcription in cancer cells, and this induces the phosphorylation of AMPK through the interaction with AMPK (8,11). According to these studies, the inhibition of mTOR activity and cell cycle arrest does not occur through AMPK phosphorylation when sestrin 2 transcription is suppressed. Thus, we hypothesized that the quercetin-induced generation of intracellular ROS and the induction of apoptosis through AMPK activation are dependent on sestrin 2 expression. Our results revealed that quercetin suppressed proliferation and induced apoptotic cell death by increasing the expression of sestrin 2. Moreover, quercetin, by increasing intracellular ROS production, can transcript sestrin 2 directly, and the upstream function in the AMPK/p38 signaling pathway was confirmed.

Importantly, it is known that approximately 50% of cancer cells are p53 mutant (25). Thus, a p53-independent manner for the induction of apoptosis is very important in cancer prevention studies. Our results revealed that not only does quercetin regulate the AMPK/p38 signaling pathway by increasing sestrin 2 expression, but it induces the apoptosis of HT-29 colon cancer cells, which are p53 mutant cells.

In this study, we demonstrate that quercetin induces apoptosis through the activation of the ROS/AMPK/p38 pathway. During this process, the increased expression of sestrin 2 induced by quercetin was found to be crucial. The silencing of sestrin 2 silencing using siRNA resulted in the deactivation of AMPK and p38 and did not reduced cell viability following treatment with quercetin. Furthermore, quercetin induced the generation of intracellular ROS and induced apoptosis by regulating the sestrin 2/AMPK/p38 pathway in p53 mutant cells. From these results, it can be suggested that the quercetin-induced apoptosis is carried out through the sestrin 2/AMPK/p38 signaling pathway, and that sestrin 2 is an important regulator of the AMPK/p38 pathway in a p53-independent manner.
